# Chemical Diversity and Ecological Origins of Anti-MRSA Metabolites from *Actinomycetota*

**DOI:** 10.3390/antibiotics14111060

**Published:** 2025-10-23

**Authors:** Sayoane Pessoa Fernandes, Luana Layse Câmara de Almeida, Thalisson Amorim de Souza, Genil Dantas de Oliveira, Marcelly da Silveira Silva, Valnês da Silva Rodrigues-Junior, Harley da Silva Alves, Samuel Paulo Cibulski

**Affiliations:** 1 Programa de Pós-Graduação em Ciências Farmacêuticas, Departamento de Farmácia, Universidade Estadual da Paraíba (UEPB), Campina Grande 58429-500, PB, Brazil; pessoasayoane@gmail.com (S.P.F.); lu.laysec@gmail.com (L.L.C.d.A.); marcelly.silva@aluno.uepb.edu.br (M.d.S.S.); 2 Laboratório Multiusuário de Caracterizaçaão e Análise (LMCA), Instituto de Pesquisa em Fármacos e Medicamentos, Universidade Federal da Paraíba (UFPB), João Pessoa 58051-900, PB, Brazil; thalisson.amorim@ltf.ufpb.br (T.A.d.S.); genil.98dantas@gmail.com (G.D.d.O.); 3 Programa de Pós-Graduação em Produtos Naturais e Sintéticos Bioativos, Universidade Federal da Paraíba (UFPB), João Pessoa 58051-900, PB, Brazil; valnesjunior@cbiotec.ufpb.br; 4 FACISA—Faculdade de Ciências da Saúde do Trairi, Universidade Federal do Rio Grande do Norte (UFRN), Santa Cruz 59200-000, RN, Brazil

**Keywords:** natural products, drug discovery, antibiotic-producing bacteria, bioprospection

## Abstract

Antimicrobial resistance (AMR) poses a major global threat to human health. Among multidrug-resistant pathogens, MRSA is a leading cause of severe nosocomial infections, urgently demanding the discovery of novel antimicrobial agents. Nature, particularly *Actinomycetota*, remains a prolific source of potent bioactive compounds to combat pathogens. This review analyzes recent advancements in anti-MRSA compounds from *Actinomycetota*. We highlight the most promising bioactive metabolites, their sources, mechanisms of action, and current limitations. Our analysis identified numerous compounds with potent activity against MRSA, including chromomycins, actinomycins, diperamycin, lunaemycin A, lactoquinomycin A, and weddellamycin, which exhibit submicromolar minimal inhibitory concentrations (MICs). The renewed interest in exploring *Actinomycetota* de novo is directly driven by the AMR crisis. Furthermore, bioprospecting efforts in underexplored ecological niches, such as mangroves and marine sediments, have proven highly promising, as these habitats often harbour unique microbial communities producing novel metabolites. These findings underscore the critical importance of ecology-driven drug discovery in expanding the antimicrobial arsenal and effectively addressing the global health challenge of MRSA and other resistant pathogens.

## 1. Introduction

The emergence and rapid spread of antimicrobial resistance (AMR) poses one of the most severe global threats to public health in the 21st century [1,2]. According to a report by the Organization for Economic Co-operation and Development (OECD), drug-resistant infections could claim the lives of approximately 2.4 million people across Europe, North America, and Australia between 2015 and 2050. In low- and middle-income countries, AMR is already at high levels and is projected to grow even more rapidly than in OECD member states. For instance, in Indonesia, Brazil, and the Russian Federation, between 40% and 60% of infections are already resistant, compared to an average of 17% in OECD countries [3].

Antibiotic resistance is regarded as one of the most pressing and alarming health challenges of our time, often referred to as a “silent pandemic”—underreported and underestimated—yet responsible for hundreds of thousands of deaths each year [4]. An economic assessment highlights its potential impact on global healthcare systems and the economy. According to the World Health Organization [5], AMR could result in additional healthcare costs of up to US$1 trillion by 2050. If current trends continue, global economic output could decline by as much as 3.8% by 2050, translating into annual losses of up to USD 3.4 trillion. Even under scenarios of effective AMR containment, the global economy is still expected to experience measurable declines, emphasizing the urgent need for coordinated interventions to mitigate both health and economic consequences [5].

*Staphylococcus aureus* stands out as one of the most clinically significant bacterial pathogens, owing to its ability to cause a wide spectrum of human diseases [6]. This Gram-positive, non-motile, non-spore-forming coccoid bacterium is known for its opportunistic behaviour and involvement in infections ranging from superficial lesions to life-threatening systemic conditions [7]. Due to its commensal nature, *S. aureus* can colonize various areas of human and animal body surfaces, such as the skin, nostrils, oral cavity, and intestines [8]. It is notably included in the ESKAPE group (*Enterococcus faecium*, *S. aureus*, *Klebsiella pneumoniae*, *Acinetobacter baumannii*, *Pseudomonas aeruginosa*, and *Enterobacter* spp.), which encompasses the leading causes of nosocomial infections because of their capacity to evade the action of antibiotics [9].

**Figure 1:**
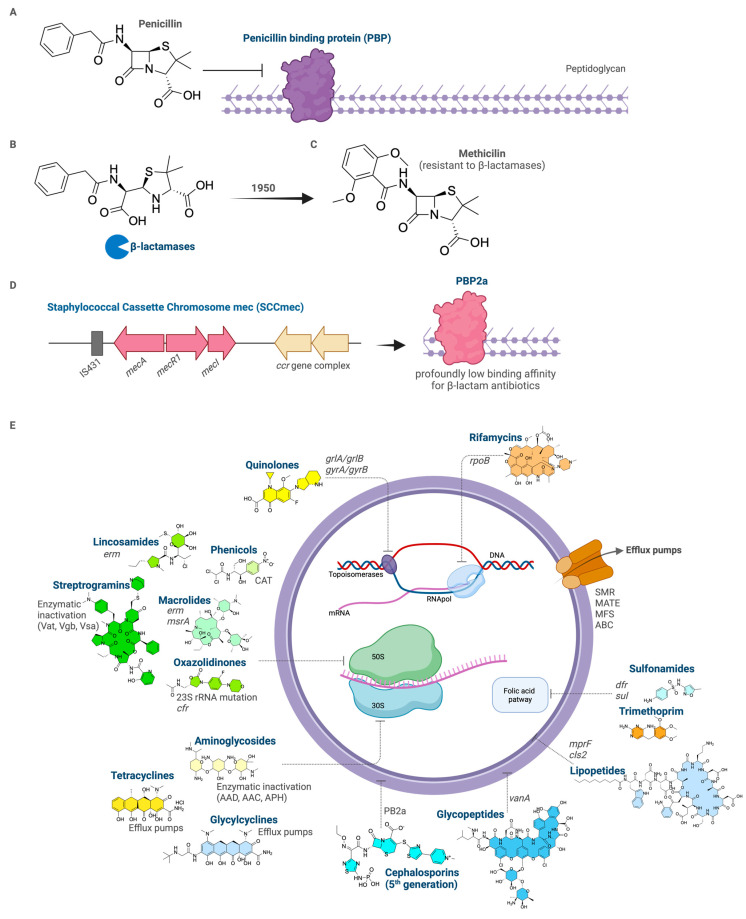
Evolution of β-lactam resistance and the molecular basis of methicillin resistance in *S. aureus.* (**A**) Penicillin inhibits cell wall synthesis by covalently binding to PBPs involved in peptidoglycan cross-linking. (**B**) β-Lactamase production emerged in *S. aureus* soon after penicillin introduction, causing hydrolysis of the β-lactam ring and drug inactivation. (**C**) Methicillin, designed to resist hydrolysis by β-lactamases. (**D**) The *mecA* gene encodes PBP2a, an alternative transpeptidase with low affinity for β-lactams. The *ccr* complex mediates SCC*mec* integration and transfer among staphylococci. (**E**) Schematic overview of the primary molecular targets of antibiotics used in the treatment of MRSA infections and the corresponding resistance mechanisms. Resistance in MRSA involves multiple adaptive strategies acting at different cellular levels: (i) target modification, notably the expression of the low-affinity penicillin-binding protein PBP2a encoded by *mecA*, which confers broad resistance to β-lactams (penicillins, cephalosporins, and carbapenems); (ii) ribosomal protection or methylation mediated by *erm* and *cfr* genes, resulting in cross-resistance among macrolides, lincosamides, streptogramins, and oxazolidinones; (iii) enzymatic inactivation of aminoglycosides, tetracyclines, and streptogramins by modifying enzymes such as AAD, AAC, APH, and Vat/Vgb/Vas; (iv) point mutations in topoisomerase genes (*gyrA, grlA, grlB*) leading to quinolone resistance; (v) mutations in the RNA polymerase β-subunit gene (*rpoB*), associated with rifamycin resistance; (vi) active efflux mechanisms mediated by multidrug transporters; (vii) cell wall target remodelling, particularly via *vanA*-type gene clusters conferring glycopeptide resistance (e.g., vancomycin); and (viii) alterations in folate biosynthesis pathways, involving *dfr* and *sul* genes that mediate resistance to trimethoprim and sulfonamides. This figure was created with BioRender.com.

Before the discovery of penicillin, *S. aureus* was one of the most feared pathogens, responsible for serious and often fatal infections. Infected wounds, abscesses, septicemia and pneumonia were common, especially during World War I. The English medical officer Alexander Fleming returned from the war and dedicated himself to studying *S. aureus*, leading to the discovery of penicillin in 1928, which revolutionized the treatment of bacterial infections (Figure 1A) [10]. Subsequently, strains of *S. aureus* emerged that could produce the β-lactamase enzyme, hydrolyzing the β-lactam ring of penicillin and rendering the antibiotic ineffective (Figure 1B). In response, methicillin was developed in 1950, designed to be resistant to these enzymes (Figure 1C). However, by the 1960s, the first cases of methicillin-resistant *S. aureus* (MRSA) began to emerge, rendering it ineffective [7]. MRSA strains demonstrate resistance to virtually all β-lactam antibiotics, as well as to other classes of antimicrobials [11].

This hallmark resistance is primarily mediated by the horizontal acquisition of the *mecA* gene, which is carried on a mobile genetic element known as the staphylococcal cassette chromosome mec (SCCmec) (Figure 1D) [12,13,14]. The *mecA* gene encodes for an alternative penicillin-binding protein (PBP), designated PBP2a. Unlike the native PBPs of *S. aureus*, PBP2a exhibits a profoundly low binding affinity for β-lactam antibiotics. The molecular basis of this resistance lies in the structure of PBP2a’s active site. In susceptible bacteria, β-lactam antibiotics act as irreversible inhibitors by forming a stable acyl-enzyme complex with the active-site serine residue of the essential PBPs, thereby halting the cross-linking of the peptidoglycan cell wall and leading to cell lysis. PBP2a, however, possesses a constricted active site pocket that sterically hinders the efficient binding of β-lactam molecules. This allows the enzyme to continue catalyzing the transpeptidation reaction necessary for cell wall biosynthesis even in the presence of otherwise inhibitory concentrations of antibiotics, thereby conferring resistance [15].

While PBP2a is the cornerstone of methicillin resistance, MRSA employs a complex arsenal of additional mechanisms to counteract virtually all major antibiotic classes (Figure 1E). These strategies are not mutually exclusive and often work in concert, leading to multidrug resistance. Key mechanisms include: (1) enzymatic inactivation of the drug (e.g., β-lactamase production in non-MRSA lineages); (2) modification of the antibiotic’s target site, as exemplified by PBP2a; (3) overexpression of multiple efflux pumps (ABC, SMR, MATE and MFS families) that actively expel antibiotics from the cell [16]; (4) reduced permeability of the cell envelope to limit drug entry; and (5) the development of alternative metabolic pathways to bypass inhibited processes [17]. Understanding this intricate battlefield between antibiotic action and the multifaceted bacterial defence systems is fundamental to appreciating the challenge of AMR and underscores the urgent need for novel therapeutic agents that can overcome these sophisticated barriers.

MRSA represents a significant global public health threat, particularly in healthcare settings, due to its high level of antibiotic resistance, which complicates the clinical management of infections [18]. According to the Centers for Disease Control and Prevention (CDC), MRSA is an increasing concern not only in hospitals but also within communities, primarily because of its ability to spread between humans and animals, high infection rates, and limited therapeutic options stemming from antimicrobial resistance [19].

Although other resistant strains, such as vancomycin-intermediate and vancomycin-resistant *S. aureus* (VISA and VRSA, respectively), also pose clinical challenges, this study focuses exclusively on MRSA. This decision is supported by its higher prevalence and greater epidemiological relevance worldwide, particularly in healthcare-associated and community outbreaks. Documented cases of VRSA remain low and geographically limited, whereas MRSA continues to be the leading cause of severe infections linked to antimicrobial resistance [20,21]. Therefore, focusing on MRSA allows for the discussion of a problem with broader magnitude and practical impact. Given this scenario, the relentless pursuit of novel therapeutic agents effective against MRSA is a medical imperative. This review aims to address this urgent need by exploring one of the most promising sources of new anti-MRSA compounds: the bioactive metabolites produced by *Actinomycetota*.

## 2. MRSA Treatment and the Antibiotic Discovery Crisis

**Figure 2:**
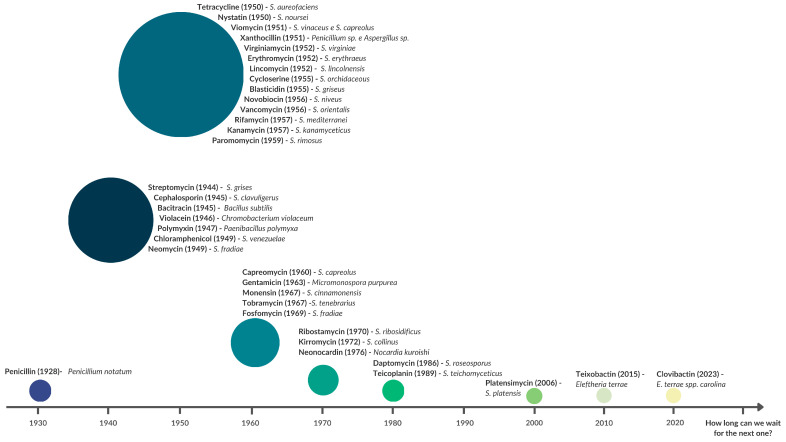
Timeline of the discovery of major antibiotics, highlighting the central role of *Actinomycetota*. The graph illustrates the period between 1928 and 2023, showcasing the “Golden Age of Antibiotic Discovery” (1940s–1960s). The size of the circles represents the relative number of discoveries per period. Most landmark antibiotics, such as streptomycin (1944), tetracycline (1950), erythromycin (1952), and vancomycin (1956), were derived from *Actinomycetota* genera, notably *Streptomyces*. The stark decline in discoveries after the 1970s emphasizes the current innovation gap, with recent rare discoveries like platensimycin (2006), teixobactin (2015) and clovibactin (2023).

The discovery of penicillin by Alexander Fleming in 1928 marked a paradigm shift in medicine, prompting universities and the pharmaceutical industry to intensify the search for new antimicrobial molecules. This effort culminated in the so-called “Golden Age of Antibiotics” (approximately 1940–1960), a period during which most major antibiotic classes currently in use were discovered [22]. A significant proportion of these groundbreaking compounds were produced by fungi and bacteria, with members of the phylum *Actinomycetota* standing out as prolific producers of secondary metabolites with potent antimicrobial activity (Figure 2).

The steep decline in the approval of new antibiotic classes after the 1970s, often termed the “discovery void”, was not a coincidence but the result of a confluence of factors. A prevailing sense of complacency emerged from the belief that the medicinal arsenal was sufficient, fueled by the success of the Golden Age which had already yielded most major antibiotic classes effective against common pathogens [22,23]. This was compounded by significant technical challenges: the frequent rediscovery of known compounds from common soil *Actinomycetota* using conventional cultivation methods led to diminishing returns, making novel discovery increasingly difficult and costly [24]. Consequently, many pharmaceutical companies deprioritized or exited antibiotic research, driven by scientific fatigue, stringent regulatory hurdles, and the poor economic returns of antibiotics, which are typically short-course therapies, unlike chronic disease medications [25]. This perfect storm led to a critical neglect of the antibiotic pipeline just as resistance mechanisms were beginning to evolve and spread globally. However, the escalating AMR crisis has now forced a paradigm shift, reigniting interest in this field. This renaissance is powered by new strategies, including the targeted exploration of underexplored ecological niches, the application of genomics to unlock “silent” biosynthetic gene clusters (BGC), and innovative cultivation techniques, paving the way for a new era of discovery [26].

The treatment of MRSA infections relies on a limited arsenal of antibiotics (Figure 1E), which includes agents such as vancomycin, daptomycin, linezolid, trimethoprim-sulfamethoxazole, quinupristin-dalfopristin, clindamycin, and tigecycline [27]. The choice of agent depends on the susceptibility profile of the isolated strain and the clinical severity of the infection. However, the progressive loss of efficacy of these antimicrobials, driven by the emergence of strains with sophisticated resistance mechanisms, has severely constrained therapeutic options [27,28].

This alarming trend is exacerbated by increasing resistance to second-line (e.g., linezolid, clindamycin) and even third-line agents (e.g., tigecycline), highlighting the rapid depletion of our antibacterial arsenal. This discovery void, evident in Figure 2 after the 1970s, underscores the urgent need for innovative strategies to combat AMR. A multifaceted approach, encompassing enhanced infection control practices in hospitals, antimicrobial stewardship to reduce unnecessary prescriptions, and the deployment of rapid diagnostic tests, is crucial. According to OECD estimates, such measures could prevent up to 1.6 million deaths by 2050 across several countries. Importantly, investing in these strategies would be cost-effective, paying for itself within a year and annual savings of approximately USD 4.8 billion [3].

## 3. *Actinomycetota*: A Prolific Source of Bioactive Natural Products

The phylum *Actinomycetota* constitutes one of the largest taxonomic groups within the domain *Bacteria* [29]. These Gram-positive bacteria are characterized by a high genomic guanine and cytosine content and often exhibit a filamentous growth morphology. While the majority are aerobic free-living organisms ubiquitously distributed in terrestrial and aquatic ecosystems, they occupy a remarkable diversity of habitats, including extreme environments such as deserts, hot springs, salt lakes, caves, and deep-sea waters [30,31,32]. This broad distribution is intrinsically linked to environmental factors, including nutrient availability, soil type, temperature, pH, and humidity, which profoundly shape their diversity and ecological function [33,34,35].

**Figure 3:**
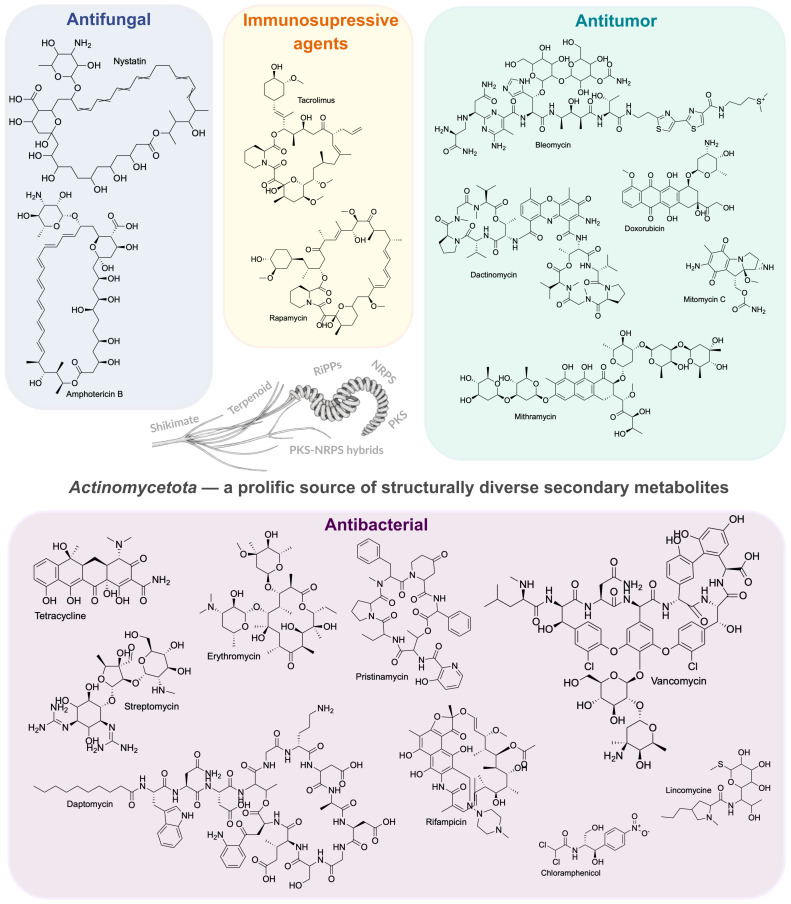
Representation of important secondary metabolites produced by *Actinomycetota*. The figure highlights the structural and functional diversity of these compounds, with applications ranging from the treatment of bacterial and fungal infections to their use as immunosuppressive and antitumor agents.

*Actinomycetota* are renowned for their high metabolic versatility, enabling them to degrade complex organic compounds such as lignin, cellulose, chitin, and hydrocarbons [36]. This capacity is facilitated by the production of diverse extracellular enzymes, pigments, and, most notably, secondary metabolites. It is this prolific secondary metabolism that positions them as a cornerstone of biotechnology. They are responsible for producing nearly 100,000 known antibiotic compounds, accounting for approximately 70% to 80% of all bioactive natural products with pharmacological or agrochemical applications [37,38]. Beyond antibiotics, their bioactivities span a wide spectrum, including antiviral (e.g., napyradiomycin A4) [39], antitumoral (e.g., mitomycin C and doxorubicin) [40,41], immunosuppressive (e.g., rapamycin) [42], and antifungal agents (e.g., nystatin and amphotericin B) [43]. Figure 3 illustrates some of these critical secondary metabolites and their clinical applications.

Given the escalating threat of antimicrobial resistance (AMR), the World Health Organization (WHO) has classified MRSA as a “high priority” pathogen, underscoring the critical need for new therapeutic agents [5]. In this pursuit, natural products remain an invaluable resource. Among them, *Actinomycetota* metabolites stand out due to their historical success and chemical diversity. This review focuses specifically on the potential of *Actinomycetota* as a source of novel antimicrobial agents against MRSA. Tapping into the vast, and still underexplored, biosynthetic repertoire of these bacteria paves the way for discovering next-generation antibiotics to combat resistant infections.

## 4. Methodology: Literature Review and Data Extraction

A literature review was conducted following a predefined protocol to identify studies reporting anti-MRSA compounds derived from *Actinomycetota*. Comprehensive electronic searches were performed across PubMed database. The core search string, adapted for each database’s syntax, was: (actinobacter* OR actinomycet* OR *Streptomyces*) AND (MRSA OR “methicillin-resistant *Staphylococcus aureus*”) AND (antimicrobial OR antibiotic OR “bioactive compound” OR “secondary metabolite”). No restrictions were placed on the publication date or language. The search was executed in February 2025, covering all records available up to that point. Additionally, the reference lists of relevant review articles and included studies were manually screened to identify additional eligible publications (a process known as snowballing).

**Figure 4:**
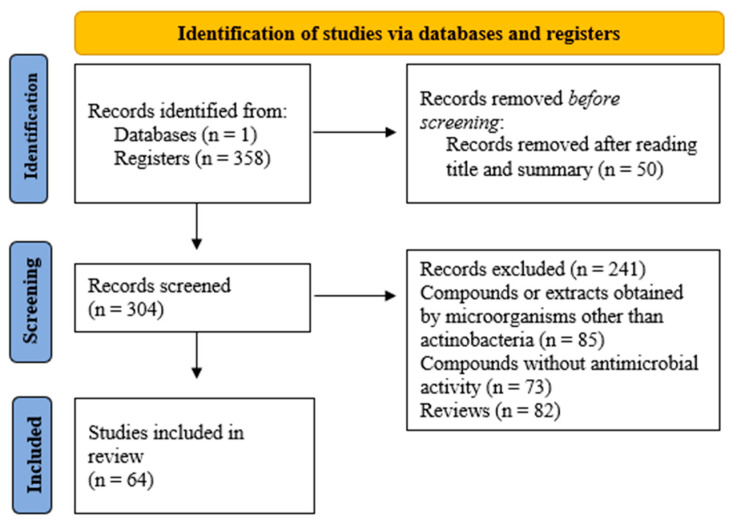
Flowchart of study selection process for the literature review. Schematic representation of the identification, screening, and inclusion of studies based on predefined eligibility criteria.

The study selection process involved two phases: screening by title and abstract and further full-text assessment. The inclusion criteria were: (1) compounds or crude extracts isolated from strains of *Actinomycetota*, (2) assessment of in vitro antimicrobial activity, (3) reported activity against MRSA, quantified by a standard metric such as MIC and, (4) original research articles. The exclusion criteria were: Studies where the active compound was derived from other microorganisms (e.g., fungi) or synthetically produced. Studies that did not test activity against MRSA specifically. Reviews, meta-analyses, editorials, conference abstracts, and patents. The study selection process is detailed in the PRISMA flow diagram (Figure 4), which outlines the number of records identified, screened, assessed for eligibility, and finally included in the review.

Data from the included studies were extracted into a standardized spreadsheet. The extracted information included: Compound Data: Name, molecular formula, molecular weight, chemical structure (drawn using ChemDraw Professional software version 23.1.2). Source Data: Genus and species of the producing actinobacterium, isolation source (e.g., soil, marine sediment, plant root), and geographic location of isolation. Biological Activity Data: MIC value (extracted in both µg/mL and µM for comparative purposes) and the specific MRSA strain(s) used in the assay. Reference Data: First author, publication year, and DOI.

## 5. Bibliometric Analysis and Source of *Actinomycetota*-Producing Anti-MRSA Compounds

**Figure 5:**
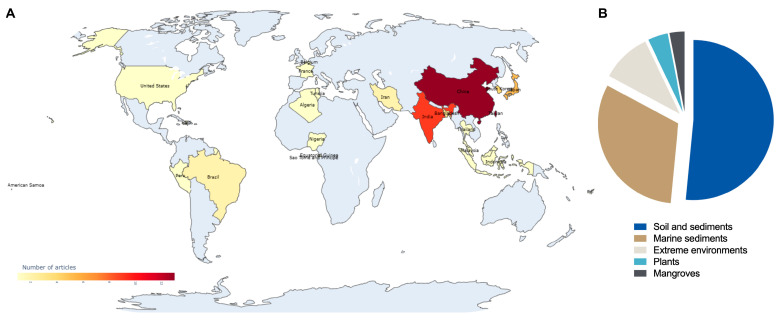
*Actinomycetota*-producing anti-MRSA metabolites. (**A**) Number of studies conducted on the isolation of anti-MRSA compounds by country. (**B**) Isolation source of the antibiotic-producing *Actinomycetota*.

The global geographical distribution of studies reporting the isolation of anti-MRSA compounds from *Actinomycetota* is presented in Figure 5A. Our analysis reveals a striking concentration of research output in Asia, which collectively accounted for approximately 77% of the included publications. This dominance underscores the region’s pivotal role in the bioprospecting of microbial natural products.

China emerged as the leading contributor, accounting for 20.3% of studies focused on anti-MRSA compounds from *Actinomycetota*. Its position as an emerging power in scientific production is driven by decades of significant strategic investments in science and technology, particularly in biotechnology and public health [44]. Japan came in second, contributing 18.8% of publications, which can be attributed to the country’s long tradition of antibiotic discovery, which originated in the post-World War II period, and the ongoing work of renowned institutions such as the Microbial Chemistry Research Foundation [45]. India ranked third, accounting for 15.6% of scientific production. This Asian prominence is in line with the conclusions of Leite et al. [45], who identified Asia as the region with the highest number of patents for antimicrobial compounds from *Streptomyces* spp., a result of public policies aimed at technological innovation and a consolidated tradition in industrial microbiology. In contrast, the representation of other continents was markedly lower. Leite et al. [45] highlighted that the Americas constituted only 4.7% of relevant patent filings, while Europe’s contribution was approximately 2%. This disparity reinforces the concept that while microbial biodiversity is globally distributed, the scientific capacity to explore it remains concentrated in nations with greater research infrastructure and funding.

This scenario highlights a critical gap and a significant opportunity. Promoting investments in biotechnology in underexplored but biodiverse regions, such as Latin America and Africa, is not just a matter of equity but a strategic imperative for drug discovery. The limited funding for science in many developing countries hampers laboratory infrastructure and access to cutting-edge technologies, ultimately constraining their potential to contribute to the global antimicrobial arsenal [46]. Therefore, fostering international collaboration and building capacity in these high-potential regions is essential to unlocking a wider range of bioactive compounds and effectively addressing the urgent threat of MRSA and other resistant pathogens.

The *Streptomyces* strains described in the included studies were isolated from a diverse range of environmental sources (Figure 5B). Soil was the main source (51.5%), followed by marine sediments (31.3%), highlighting the importance of these environments as reservoirs of microorganisms that produce bioactive compounds. Other sources included extreme environments (10.1%), plant-associated (4%), and mangroves (3%), highlighting the ecological diversity targeted by bioprospecting efforts.

The predominance of terrestrial sources, particularly soil, reflects its historical role as the primary matrix in microbiological research rather than an inherently greater biotechnological potential. *Actinomycetota* can represent up to 50% of the total bacterial population in soil, justifying their ecological and applied relevance [47]. Although the rate of novel *Streptomyces* isolation from terrestrial samples may be decreasing, a significant number of new secondary metabolites continue to be discovered in this environment [48].

In recent decades, marine sediments have gained prominence as a highly promising source. This shift is driven by the development of improved isolation techniques and a growing scientific interest in underexplored niches. The genetic and metabolic diversity in these ecosystems contributes to the discovery of novel bioactive compounds, often with equal or greater potential than those from terrestrial sources [49]. The oceans, covering 70% of the Earth’s surface and harbouring an estimated 87% of its biodiversity, contain millions of undescribed microorganisms, many residing in deep-sea sediments [50]. Marine *Actinomycetota* have evolved unique biosynthetic pathways in response to adaptive pressures like extreme pH, high pressure, and temperature fluctuations, leading to metabolites with high biotechnological potential [51,52].

Despite this potential, knowledge of marine *Streptomyces* biodiversity remains limited due to the complexity and inaccessibility of deep-sea ecosystems [53]. Beyond sediments, these microorganisms have been isolated from marine sponges, algae, corals, and fish [54], demonstrating their broad distribution and reinforcing their promise as a source of innovative natural products [55,56].

*Streptomyces* isolated from extreme environments, characterized by high salinity, extreme pH, nutrient scarcity, and temperature extremes, represented approximately 10% of the anti-MRSA compounds identified. These harsh conditions impose strong selective pressures, favouring highly adaptable microorganisms with remarkable physiological plasticity [57,58]. The recent surge in research focused on these habitats is driven by the identification of new species producing metabolites with potent biological activity, underscoring the importance of exploring inhospitable environments to expand the available bioactive compound repertoire [59].

To a lesser extent, *Streptomyces* have been isolated from plants (often as endophytes) and mangroves. In these habitats, they frequently establish mutualistic relationships, developing adaptive strategies that include the production of bioactive metabolites [60]. Endophytic *Streptomyces* can promote plant growth and produce compounds with therapeutic potential [61]. Mangroves, recognized as rich yet underexplored microbial habitats [53] are particularly promising.

Our analysis encompassed approximately 67 distinct strains of *Streptomyces* and other *Actinomycetota*-producing anti-MRSA compounds. Notably, only about 23 (~34%) were identified at the species level (Table 1 and Table 2). Most studies (~60%) designated isolates only as “*Streptomyces* sp.” This taxonomic imprecision is attributed to the high complexity of the genus, characterized by significant genomic plasticity, frequent genetic recombination, and phenotypic overlap between closely related strains. The limited resolution of 16S rRNA gene sequencing for discriminating within *Streptomyces* often makes it insufficient for accurate speciation. Many isolates also lack well-annotated reference genomes in public databases.

Accurate taxonomic identification of *Actinomycetota* strains is a cornerstone of antibiotic discovery, as it enables the unambiguous correlation of a bioactive compound with its producer organism and is crucial for comparative genomics studies. However, the limited resolution of the 16S rRNA gene for discriminating between closely related species, especially within the genus *Streptomyces*, often leads to ambiguous identifications, a recurrent problem in bioprospecting studies. To overcome this barrier, a polyphasic approach combining complementary techniques has become indispensable. Matrix-Assisted Laser Desorption/Ionization Time-of-Flight Mass Spectrometry (MALDI-TOF MS) emerges as a rapid and low-cost screening tool. However, its efficacy is directly limited by the quality and comprehensiveness of the reference database. As demonstrated in our survey, the scarcity of spectral profiles for *Actinomycetota* in commercial libraries severely restricts its ability to provide reliable identification beyond the genus level for a wide range of environmental isolates, often resulting in inconclusive identifications [126]. Therefore, for definitive taxonomic resolution, genomic techniques are essential. Average Nucleotide Identity (ANI) calculates the average percentage of identity between whole genomes, serving as a digital gold standard for species demarcation, while Multilocus Sequence Analysis (MLSA) relies on the sequences of multiple housekeeping genes to build robust phylogenies. The strategic application of these techniques, using MALDI-TOF MS for initial screening and dereplication, but relying on ANI/MLSA for the final characterization of promising strains, ensures correct and reproducible identification. This integrated approach not only prevents the rediscovery of known species but also, by providing accurate classification, facilitates the targeted mining of BGCs in genomes, accelerating the discovery of new antibiotics from taxonomically unique and well-characterized strains [127,128,129].

## 6. Anti-MRSA Secondary Metabolites from *Actinomycetota*: A Chemical Overview

This review consolidates data on 177 distinct compounds derived from *Actinomycetota*, predominantly from the genus *Streptomyces*, with demonstrated activity against MRSA. Table 1 and Table 2 form the core of this analysis, providing a comprehensive summary of these promising secondary metabolites.

To systematically organize this chemical diversity and provide insight into their biosynthetic origins, the compounds have been classified according to their major metabolic pathways. This approach underscores the fact that bioactivity is a direct manifestation of underlying genetics and biochemistry. Among the most significant pathways identified are: Non-Ribosomal Peptide Synthesis (NRPS) and Polyketide Synthase (PKS) pathways. These pathways are renowned for generating metabolites with complex chemical architectures and potent, diverse biological activities, accounting for a majority of the clinically used antibiotics. Additionally, promising crude extracts and partially characterized compounds are summarized separately in Table 2.

This classification highlights the richness of BGCs within *Actinomycetota* genomes, particularly in *Streptomyces*. The observed structural variety results from extensive metabolic versatility, driven by evolutionary mechanisms such as gene duplication, horizontal gene transfer, and genomic rearrangements [28,130,131,132]. Thus, organizing compounds by their biosynthetic logic not only provides a systematic framework but also emphasizes the vast, untapped potential of *Actinomycetota* for discovering novel therapeutic agents.

### 6.1. Non-Ribosomal Peptides

Non-ribosomal peptides (NRPs) represent a critically important class of secondary metabolites, predominantly produced by bacteria and fungi. Unlike ribosomal peptides, NRPs are synthesized by large, modular enzyme complexes known as non-ribosomal peptide synthetases (NRPSs), which function independently of the ribosome [133].

Each NRPS module is responsible for activating, modifying, and incorporating a single amino acid (or other building block) into the growing peptide chain. This assembly-line process allows for the incorporation of over 500 different non-proteinogenic amino acids, D-amino acids, and other organic acids, leading to an enormous diversity of structures that are inaccessible to the ribosomal machinery [134]. The structural complexity of NRPs results in diverse and potent biological activities, making them valuable for drug discovery [135].

**Figure 6:**
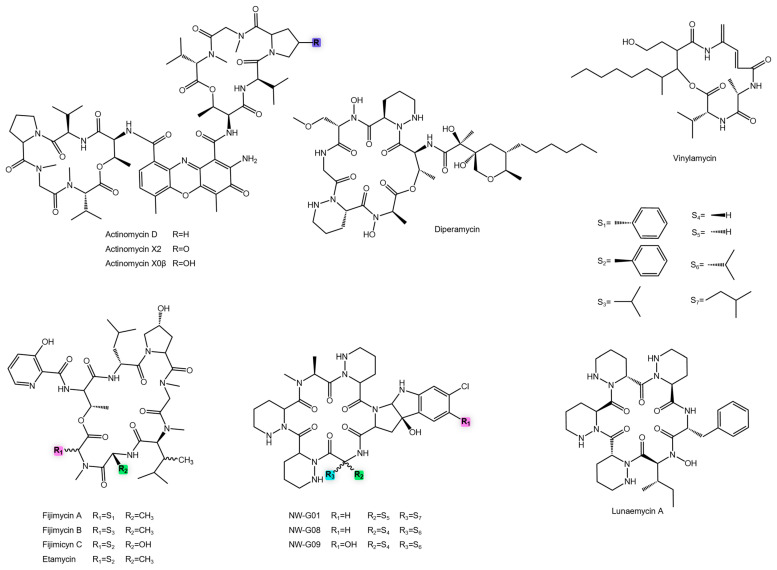
Chemical structures of NRP with anti-MRSA activity.

Notable anti-MRSA compounds from this class include last-resort antibiotics like daptomycin and other structurally complex peptides. Table 1 presents a detailed list of *Actinomycetota*-derived NRPs and other metabolites exhibiting potent activity against MRSA, including their producing organisms, MIC values, and chemical characteristics. In addition, Figure 6 shows all NRPs with anti-MRSA activity discussed in this study.

Cyclodepsipeptides are a prominent group of NRPs characterized by the replacement of one or more amino acids with hydroxy acids, introducing ester bonds into their macrocyclic ring [136,137]. This structural feature confers remarkable diversity and a broad spectrum of biological activities, including antitumor, antifungal, and antibacterial properties [138,139,140]. The ability of α-hydroxy acids to mimic amino acids allows for interaction with diverse protein targets. Furthermore, the cyclic conformation and frequent N-methylation enhance stability against hydrolytic enzymes, improving potential oral bioavailability [141].

Actinomycins, discovered by Waksman and Woodruff [142], are a classic class of chromopeptide antibiotics produced by *Streptomyces* spp. Structurally, they consist of a phenoxazinone chromophore linked to two cyclic pentapeptide lactone chains. Over 40 analogues have been described, with variations in the peptide units dictating their functional diversity [143,144]. Although renowned as chemotherapeutics, actinomycins exhibit potent activity against Gram-positive bacteria. Actinomycin D, V, X_0_β, and X_2_ have demonstrated exceptional efficacy against MRSA, highlighting their repurposing potential [62,63,64,65,143]. This broad-spectrum bioactivity stems from their canonical mechanism of action: the inhibition of RNA transcription. The planar phenoxazinone chromophore of actinomycins intercalates into bacterial DNA, physically impeding the progression of RNA polymerase and halting gene expression. While this mechanism underpins their remarkable potency, it is also responsible for their significant cytotoxicity in eukaryotic cells, which remains the primary barrier to their systemic antimicrobial use. Strategies such as structural modification for selective targeting or formulation for topical application could potentially mitigate this limitation and unlock their value as anti-MRSA agents [144].

Fijimycins and etamycin are cyclic depsipeptides that share a macrocyclic core with thiazoline rings and non-proteinogenic amino acids. Fijimycin A and etamycin A displayed the greatest antibacterial potency, showing approximately an 8-fold increase in activity relative to fijimycin B and a 2-fold increase compared to fijimycin C. The significant activity drop in fijimycin B underscores how subtle structural changes (hydroxyl and methyl group positioning) dramatically affect target interaction and cellular penetration [66,145].

Further illustrating the potency of this class, diperamycin outperformed vancomycin against MRSA [67]. Similarly, vinilamycin showed promising activity [68]. The recently identified cyclic hexapeptide lunaemycin A exhibits remarkable potency (MIC 0.17 µM), suggesting an adaptive function in microbial competition and emphasizing the value of underexplored habitats as sources of new antibiotic leads [69].

Finally, the depsipeptides NW-G01, NW-G08, and NW-G09, isolated from the terrestrial *Streptomyces alboflavus*, exhibited potent activity against clinical MRSA isolates. Among them, NW-G08 was the most active, further illustrating how subtle structural modifications can markedly impact antibacterial potency [70,71].

### 6.2. Polyketides and Other Metabolites

Polyketides represent one of the most prolific families of microbial secondary metabolites, biosynthesized by large multimodular enzyme complexes known as polyketide synthases (PKS). These enzymes assemble their products from simple fatty acid precursors through a process mechanistically analogous to fatty acid synthesis, yielding an immense structural diversity that includes macrolides, aromatic polyketides, polyethers, and pyranones [146]. This chemical diversity underpins a wide range of therapeutic applications, with iconic examples including the antibiotic erythromycin, the antifungal amphotericin B, the antiparasitic avermectin, and the chemotherapeutic agent doxorubicin [147].

**Figure 7:**
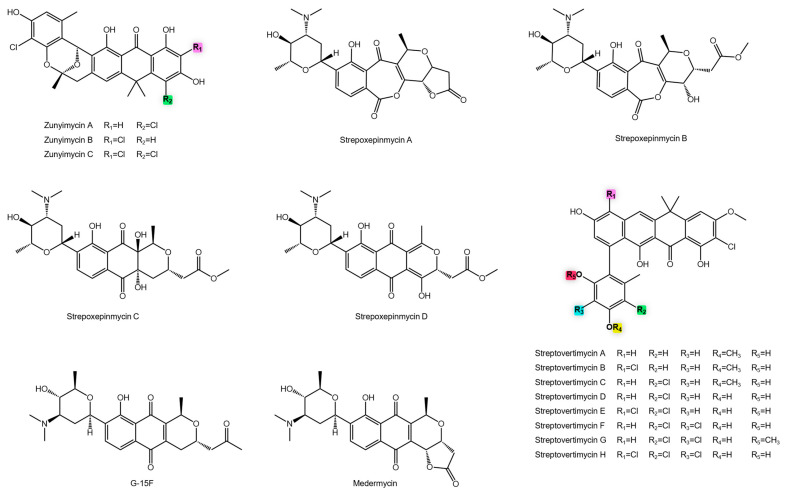
Chemical structures of anti-MRSA metabolites.

Our analysis identified several aromatic polyketides with promising activity against MRSA. Among them, zunyimycins A, B and C, isolated from *Streptomyces* sp. FJS31-2 (Figure 7). Zunyimycin C emerged as the most potent analogue, underscoring how subtle structural modifications on the polyketide scaffold can significantly enhance antibacterial efficacy [72].

Medermycin and its analogue G-15F (Figure 7) further exemplify structure–activity relationships among anthracycline-type antibiotics. Despite lacking the methoxyglycosyl group, G-15F remains only two-fold less potent than medermycin, indicating that glycosylation enhances, but is not essential for bioactivity [73,148].

The marine strain *Streptomyces* sp. XMA39 proved to be a rich source of aromatic polyketides, producing the known medermycin alongside new strepoexpimicins A–D (Figure 7). The strepoexpimicins showed a wide range of activities (MIC 6.36 to 29.67 µM), with the potency order (D > C > A > B) providing a clear example of how minor structural alterations dictate bioactivity. The stark >12-fold difference in potency between medermycin and strepoexpimicin D underscores the profound impact of specific functional groups on antibacterial efficacy, likely influencing target binding, membrane permeability, or susceptibility to efflux pumps [74,149].

**Figure 8:**
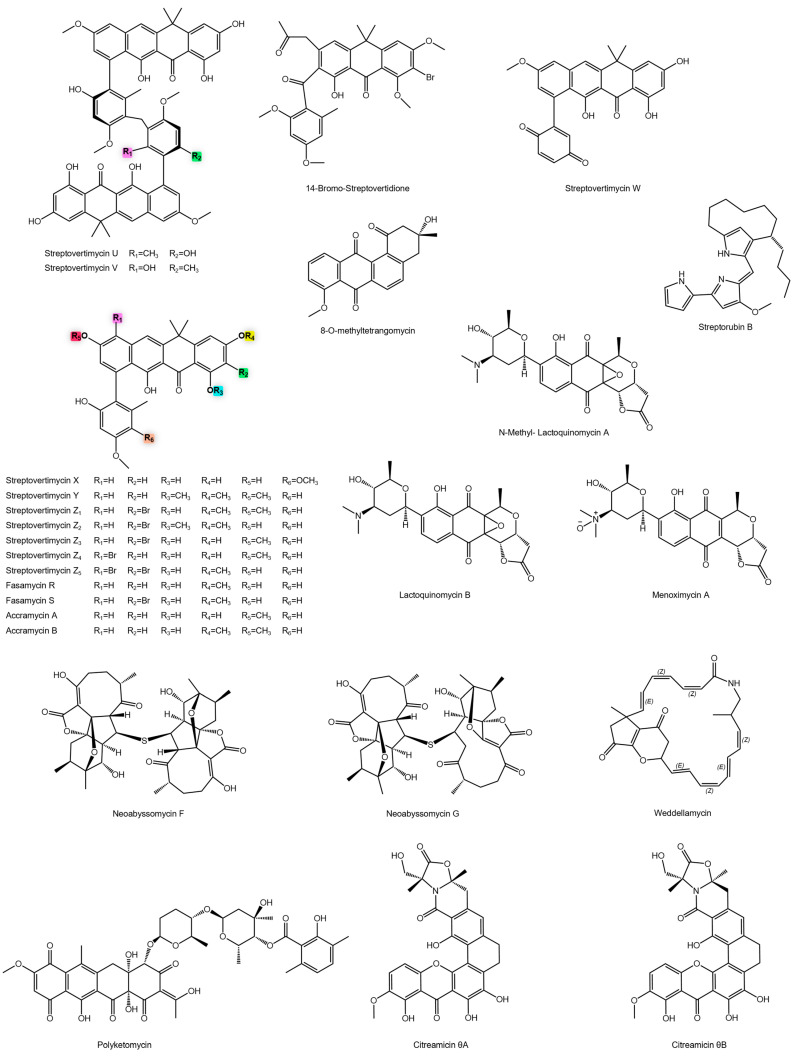
Chemical structures of anti-MRSA metabolites.

The biosynthetic potential of soil-derived *Actinomycetota* was further demonstrated by *Streptomyces morookaense* SC1169, which produces a vast array of fasamycin-type aromatic polyketides, including the streptovertimycins (A–H, U–Y, Z1–Z5), fasamycins R and S, and accramycins A and B [75,76] (Figure 8). This collection of analogues served as a powerful toolkit for structure-activity relationship (SAR) studies. Potency varied dramatically, from the highly active fasamycin S and streptovertimycin G to inactive compounds (Figure 7). This spectrum of activity confirms that subtle variations, such as methylation, hydroxylation, halogenation, and the presence of specific aromatic side chains, are critical determinants of molecular interactions with bacterial targets like topoisomerases or cell wall biosynthesis enzymes [150].

In addition, streptorubin B (Figure 8), an aromatic polyketide, was evaluated against a resistant clinical strain of MRSA. The compound displayed limited efficacy, with a high MIC [77]. In contrast, 8-*O*-methyltetrangomycin (Figure 8) showed an MIC 13 times lower [78]. This difference is likely due to structural modifications such as methylation at position 8 of the anthracenoquinone, which can enhance lipophilicity, chemical stability, or target affinity.

Another noteworthy group of polyketides was identified from *Streptomyces bacillaris* MBTC38, isolated from marine sediments. This strain produced four analogues: Lactoquinomycin A-B, *N*-methyl-lactoquinomycin A, and menoxymycin A (Figure 8) [79]. Among them, lactoquinomycin A exhibited high activity, with MIC values as low as 0.13 µM, surpassing many clinically used antibiotics (Figure 8). *N*-methyl-lactoquinomycin A also showed potent activity, suggesting that methylation of the amino-glycosidic ring enhances target affinity or membrane permeability. Menoxymycin and lactoquinomycin B displayed moderate yet still clinically relevant potency. Initially, the mechanism of action was hypothesized to involve disruption of the bacterial membrane potential, based on the structural similarity of lactoquinomycin A to compounds like γ-actinorhodin [151]. However, experimental validation using the membrane-potential-sensitive dye DiSC_3_(5) ruled out this mechanism, as no membrane depolarization or permeabilization was observed. Instead, lactoquinomycin A was found to damage bacterial DNA by intercalating into the double helix and inducing relaxation of supercoiled plasmid DNA, a mechanism reminiscent of classical intercalators like doxorubicin [79].

Neoabyssomycins F and G (Figure 8) exhibited moderate activity against MRSA isolates. Despite their lower potency, their unusual macrocyclic structures suggest novel mechanisms of action and provide promising scaffolds for antibiotic development [80]. Notably, the abyssomicin class, such as abyssomicin C, exhibits its anti-Gram-positive activity through a distinct mechanism: the inhibition of the p-aminobenzoic acid (PABA) biosynthesis pathway by targeting the enzyme PabB, thereby depleting essential folate cofactors [152].

Within the macrolactam subclass (PML), weddellamycin (Figure 8) stands out. Isolated from *Streptomyces* sp. DSS69, obtained from an Antarctic sponge, it showed remarkable potency [81]. PMLs are natural products characterized by 16–34-membered macrolactam rings containing distinct polyene fragments, formed through unusual biosynthetic processes such as the incorporation of β-amino acids and transannular rearrangements [153,154]. Their structural complexity underlies a broad spectrum of bioactivities, including antiviral, antibacterial, antifungal, and antitumor effects [155,156,157,158,159,160].

**Figure 9:**
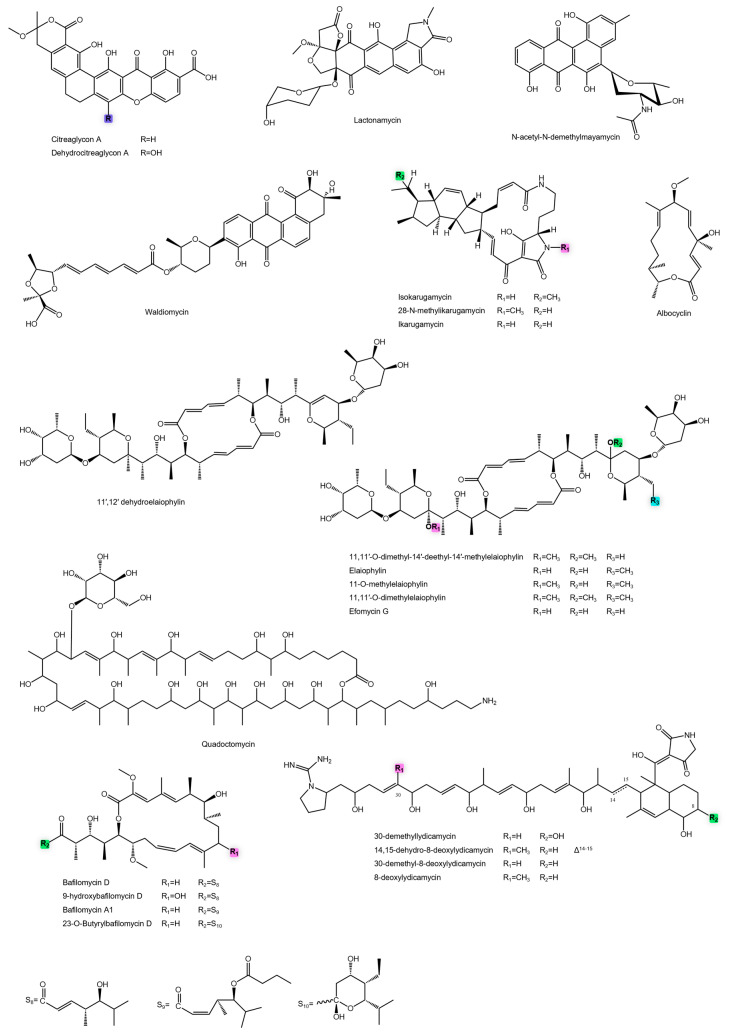
Chemical structures of anti-MRSA metabolites.

Polyketomycin (Figure 8) also exhibited remarkable antibacterial potency, ranking among the most active aromatic polyketides reported to date [82]. Its tetracyclic naphthoquinone scaffold, with strategically positioned hydroxyl and oxo groups, facilitates DNA intercalation and disruption of vital bacterial processes. Similarly, *Streptomyces caelestis* produced citreamycin θA, citreamycin θB (Figure 8), and dehydrocitreaglycon A (Figure 9), all with strong activity [83]. In contrast, citreaglycon A showed weaker potency (Figure 9). The activity differences can be attributed to variations in oxidation state and functional group distribution, with highly oxidized structures (quinones, hydroxyls) promoting stronger DNA and enzyme interactions [161,162]. Additional potent metabolites include lactonamycin from *Streptomyces rishiriensis* MJ773-88K4 [84], while others such as *N*-acetyl-*N*-demethylmayamycin [85] and waldiomycin [86], showed more moderate activity (Figure 9).

Polycyclic polyketides also exhibited notable antibacterial activity. Isoikarugamycin and ikarugamycin showed comparable potency, whereas 28-N-methylikarugamycin (Figure 9) was approximately two-fold more active, suggesting that methylation may enhance bacterial target interactions [87].

Macrolides and glycosylated macrolides further enrich this chemical space. Albocycline (Figure 9) showed potent activity, linked to its α,β-unsaturated conjugated macrocyclic structure, which enables covalent inhibition of MurA, a key enzyme in peptidoglycan biosynthesis [88,163].

**Figure 10:**
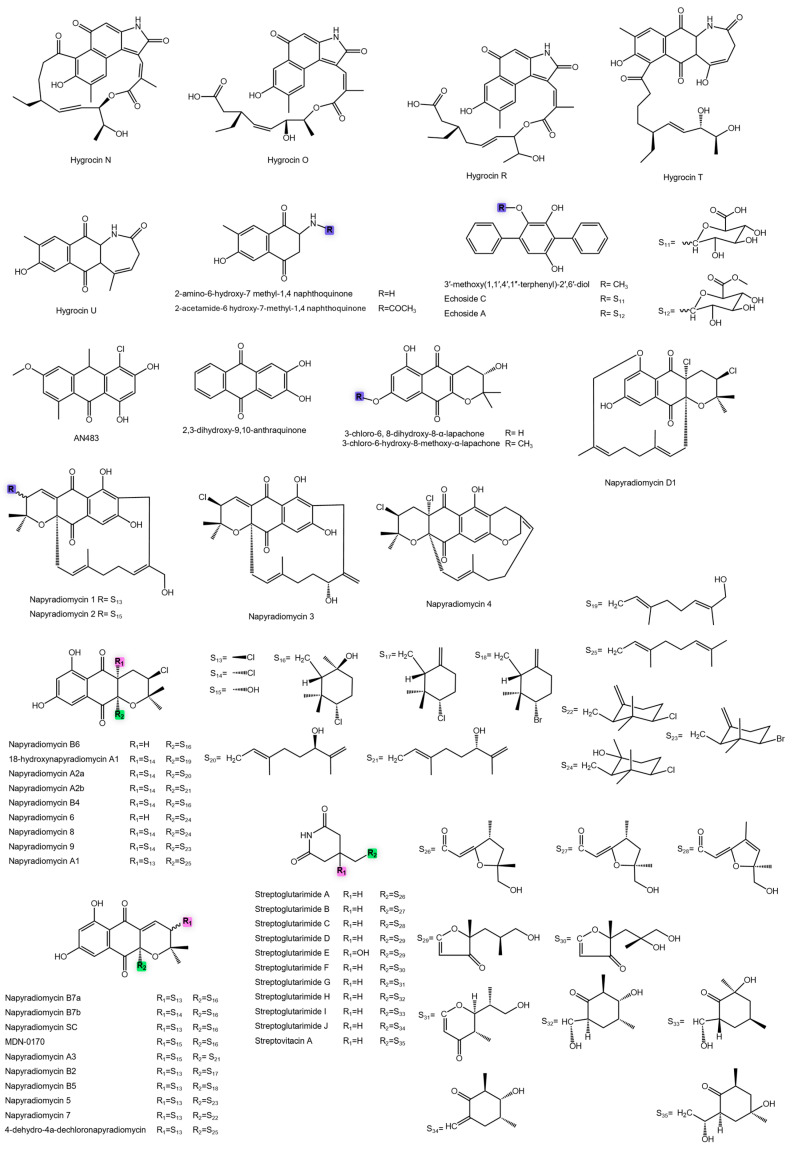
Chemical structures of anti-MRSA metabolites.

Marine-derived *Streptomyces* sp. 7–145 produced elaiofilin and related glycosylated macrolides (Figure 9). Elaiofilin retained high potency, as did 11′,12′-dehydroelaiofilin and 11-*O*-methyllaiofilin. In contrast, heavily methylated analogues such as 11,11′-*O*-dimethyl-14′-deethyl-14′-methylelaiophylin (Figure 9) showed drastically reduced activity, likely due to increased lipophilicity, reduced solubility, and steric hindrance [89]. Complementing these metabolites, efomycin G (MIC 1.98 µM) from the same strain reinforced the potential of marine-derived *Streptomyces* as sources of potent antibiotics (Figure 10).

Within the group of macrocyclic polyketides, quadoctomycin (Figure 9), produced by *Streptomyces* sp. MM168-141F8, exhibited potent activity with submicromolar MIC values [90]. Its highly functionalized macrocyclic scaffold likely facilitates efficient interactions with bacterial targets, making it a promising candidate for therapeutic development.

In comparison, *Streptomyces platensis* TP-A0598, highlights how subtle structural variations modulate bioactivity. Among its lidicamycin analogues, the derivative 30-demethyl-8-deoxylidicamycin displayed the strongest effect. Related congeners such as 30-demethyllidicamycin and 8-deoxylidicamycin maintained good potency, while 14,15-dehydro-8-deoxylidicamycin was less active [91] (Figure 9). These results underscore the importance of targeted modifications, such as demethylation or deoxygenation, in fine-tuning antibacterial efficacy.

Further examples include macrolide polyketides from *Streptomyces* sp. HZP-2216E. This strain produced bafilomycin D, 9-hydroxybafilomycin D, bafilomycin A1, and 23-*O*-butyryl bafilomycin D (Figure 9) [92]. The most potent analogue, 23-*O*-butyryl bafilomycin D, illustrates that acyl substitutions can enhance activity. Nonetheless, these values remain modest compared to front-line antibiotics with MICs below 1 µM, highlighting the need for further structural or pharmacological optimization.

**Figure 11:**
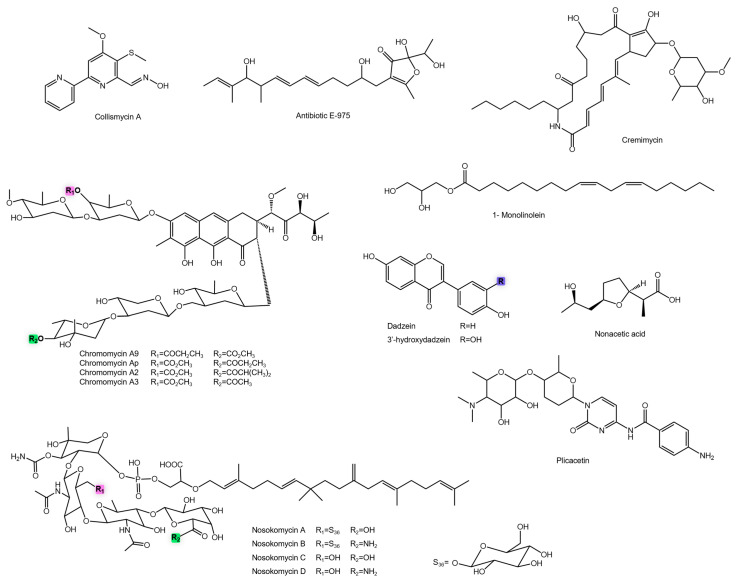
Chemical structures of anti-MRSA metabolites.

The mangrove-derived *Streptomyces* sp. ZZ1956 also demonstrated a rich biosynthetic capacity, producing diverse polyketides and quinones with variable anti-MRSA activity [93]. Among the hygrocins, hygrocin U and R showed moderate potency, whereas hygrocin N and O were less effective, and hygrocin T displayed minimal activity (Figure 11). In the quinone series, 2-amino-6-hydroxy-7-methyl-1,4-naphthoquinone exhibited weak activity, while echosides A and C were moderate (Figure 10).

By contrast, quinones from terrestrial *Streptomyces* strains displayed even lower efficacy. For instance, AN483 (Figure 10) from *Streptomyces* sp. AN100483 showed an MIC of 100.41 µM, while 2,3-dihydroxy-9,10-anthraquinone (Figure 10) from *Streptomyces* galbus ERNLG-127 had an MIC of 52.07 µM [94,95]. These data suggest that while macrocyclic scaffolds and selective substitutions can yield highly active analogues, simpler quinonoid structures often exhibit only moderate to weak activity.

The *Streptomyces* sp. CA-271078 strain, biosynthesizes a wide range of naphthoquinone-type meroterpenoids, particularly structural variants of napyradiomycin. These compounds displayed diverse antimicrobial activities, with MIC values ranging from >216.95 to 6.29 µM, clearly demonstrating the influence of structural modifications on biological efficacy [96]. Compounds such as napyradiomycin A3, B7b, and SC showed weak activity, suggesting that certain structural configurations may hinder interaction with bacterial targets (Figure 10). Similarly, MDN-0170, napyradiomycin B6, 18-hydroxynapyradiomycin A1, 3-chloro-6,8-dihydroxy-8-α-lapachone, and hydroxy-8-methoxy-α-lapachone reinforced this pattern, indicating that hydroxylation or other substitutions at specific sites can diminish activity. In contrast, napyradiomycin D1 and variants A2a and A2b (Figure 10) exhibited stronger activity, suggesting that a balanced interplay between hydrophobicity and functional groups promotes bacterial target interaction. Compounds B4 and B5 displayed intermediate but consistent activity within a more conserved structural framework. The standout was napyradiomycin B2, whose potent activity likely reflects an optimal structural arrangement for binding bacterial targets [96] (Figure 10). Overall, these findings highlight the strong relationship between chemical structure and antimicrobial activity in naphthoquinone-type meroterpenoids, showing that even subtle modifications, particularly hydroxyl or halogen insertions, can markedly affect bioactivity [96].

Additional napyradiomycin derivatives (1–6, B2–B4) (Figure 10) were isolated from *Streptomyces* sp. *CNH-070*, obtained from marine sediments, which also exhibited wide variability in activity [97]. Napyradiomycins 3–6 and napyradiomycin 2 showed weak antibacterial activity (MICs > 120 µM), whereas napyradiomycin 1 exhibited moderate potency (MIC 34.92 µM). The most active analogue, napyradiomycin B3, was about ten-fold more potent, suggesting an optimized structural configuration for bacterial target binding. Napyradiomycins B2 and B4 were less active but remain valuable scaffolds for future derivatization.

Within the naphthoquinone-type meroterpenoids, MDN-0170 and 3-chloro-6,8-dihydroxy-8-α-lapachone (Figure 10) showed low activity. In contrast, napyradiomycin A1 was exceptionally potent (MIC 0.5–1 µg/mL), suggesting a structural arrangement highly compatible with bacterial target binding. Its analogue, 4-dehydro-4a-dechloronapyradiomycin A1 (Figure 10), also displayed activity, reinforcing the idea that small alterations, such as dechlorination or dehydrogenation, significantly affect bioactivity [98].

Beyond the naphthoquinones, glutarimide-class metabolites, including streptogutarimides A–J and streptovitacin A (Figure 10), exhibited moderate activity [99]. Their uniform activity suggests a conserved bioactive core, though their potencies remain above the threshold typically required for clinical application (<10 µM). Nevertheless, their simpler synthetic profiles may facilitate structural optimization.

Other notable compounds include colismycin A (Figure 11), which showed moderate efficacy against MRSA [63]. While not highly potent, its unusual insect-associated origin emphasizes the importance of ecological diversity in natural product discovery. Similarly, antibiotic E-975 (Figure 11), from terrestrial *Streptomyces* sp. AT37, displayed low-to-moderate activity [100].

Cremimycin (Figure 11), a metabolite isolated from *Streptomyces* sp. MJ635-86F5, exhibited remarkable activity against MRSA strains, with submicromolar MIC values [101]. This potency places cremimycin among the most effective compounds identified in this survey. Its strong antimicrobial performance, combined with its terrestrial origin and specificity toward Gram-positive pathogens, underscores its strategic importance in the search for new agents against multidrug-resistant bacteria. Moreover, its unusual peptide-based structure, linked to modified ribosomal biosynthesis, confers distinctive pharmacological features, including enhanced stability and selectivity [101].

Compounds from the chromomycin class also demonstrated exceptional efficacy. Chromomycin A2, A3, A9, and Ap (Figure 11) belong to the aureolicin-type aromatic glycosides, characterized by a polycyclic scaffold attached to deoxyglycan sugar chains [102]. These compounds displayed MIC values ranging from 0.05 to 0.11 µM, with chromomycin Ap and A2 being the most potent. Such low MICs suggest strong target affinity, structural stability, and efficient cell penetration, desirable traits for clinical antibiotic development (Figure 11). Chromomycins A3 and A9 also maintained excellent activity, only slightly less potent than A2 and Ap. The consistent results across all chromomycin derivatives reinforce that their core polycyclic skeleton provides intrinsic antibacterial potency, with side-chain variations primarily modulating selectivity and toxicity. This observation agrees with previous studies on aureolicins, which show that sugar-chain modifications rarely diminish the central DNA-binding activity mediated through minor groove interactions [164]. Chromomycin A_3_ exerts its potent antibacterial and antitumor effects by selectively inhibiting DNA-dependent RNA synthesis [165]. The compound acts as a potent inhibitor of RNA polymerase, a mechanism analogous to that of actinomycin D. Compared with other classes examined in this study, including aminoglycosides, naphthoquinones, and non-ribosomal peptides, chromomycins stand out as the most potent compounds by absolute MIC values.

By contrast, *Streptomyces* sp. YBQ59 produced metabolites with only moderate to weak activity. For example, 1-monolinolein and bafilomycin D displayed MIC values of 23.98 µM and 18.35 µM, respectively, while nonanoic acid and the isoflavonoids daidzein and 3′-hydroxydaidzein showed even lower efficacy (Figure 11) [103].

Among the moenomycins, nosokomycins A–D (Figure 11) displayed activity against MRSA but with high MIC values, indicating weak potency [104]. The similarity of these values suggests that peripheral structural modifications among nosokomycins had little effect on antimicrobial performance, reflecting a highly conserved bioactive core. Unlike other polyketide subclasses, such as zunimycins, where minor structural changes produced notable shifts in potency, nosokomycins may require more extensive molecular redesign to enhance activity [166,167].

Finally, the disaccharide nucleoside antibiotic plicacetin (Figure 11) showed relevant anti-MRSA activity [105]. As an aminoglycoside derivative, a class already established in clinical antibacterial therapy, plicacetin holds promise as a scaffold for developing new analogues with improved selectivity and reduced toxicity, warranting further preclinical exploration.

### 6.3. Promising Anti-MRSA Activity in Crude Extracts and Partially Purified Fractions

Beyond the fully characterized compounds detailed in Table 1, our survey underscores a critical and often underutilized resource: the substantial anti-MRSA potential residing in crude extracts and partially purified fractions from *Streptomyces* (Table 2). These findings are not merely preliminary data but represent a direct pipeline to future antibiotic discovery, highlighting specific microbial strains and ecological niches that demand prioritized investigation.

The compiled data reveal a clear trend regarding extraction efficacy. Ethyl acetate and methanolic extracts consistently yielded the most potent activities, suggesting their superior ability to concentrate bioactive secondary metabolites with anti-MRSA properties. This is powerfully demonstrated by several extracts exhibiting MIC values rivalling those of purified antibiotics (≤2 µg/mL), indicating the presence of highly potent compound(s) within these complex mixtures.

Furthermore, the ecological patterns observed among the producing strains are striking. A significant proportion of the most active extracts were derived from actinomycetes isolated from marine sediments and mangroves [107,112,120,121,124,167]. This reinforces the paradigm that underexplored and competitive environments are rich reservoirs of microbial strains equipped with novel defensive chemistries.

## 7. Concluding Remarks

This comprehensive review consolidates evidence on the immense potential of *Actinomycetota* as a source of novel therapeutics against the global threat of MRSA. Our analysis of 177 secondary metabolites unequivocally demonstrates that this phylum, and particularly the genus *Streptomyces*, continues to produce compounds with remarkable potency against multidrug-resistant strains. The identification of agents such as chromomycins A_2_ and Aₚ, actinomycin V, lactoquinomycin A, and weddellamycin, which exhibit exceptional sub-micromolar activity, underscores a far-from-exhausted reservoir of chemical diversity.

A critical finding of this analysis is the direct link between ecological sourcing and chemical novelty. The most promising anti-MRSA compounds were frequently isolated from underexplored and extreme environments, including marine sediments, mangroves, and caves. This confirms that the strategic, ecology-driven exploration of microbial communities is a paramount and productive strategy for circumventing the rediscovery of known compounds from common sources.

In conclusion, the chemical arsenal derived from *Actinomycetota* represents a vital and robust pipeline in the fight against AMR. The potent activities documented herein provide a compelling argument that the pursuit of novel antibiotics from these prolific producers must remain a central pillar of biomedical research. The continued investigation of their biosynthetic capacity is not a return to the past, but a necessary pathway to a more secure antimicrobial future.

## 8. Future Prospects

The compelling evidence of anti-MRSA potential within *Actinomycetota*, as outlined in this review, provides a robust foundation for future research. To translate this potential into novel therapeutics, the field must embrace a synergistic, technology-driven approach that moves beyond traditional methods. The following interconnected strategies represent the most promising avenues for unlocking the next generation of antibiotics:

I. Genomics-Guided Discovery and Activation of Silent BGCs

A significant portion of the biosynthetic potential of *Actinomycetota* remains untapped within silent or cryptic BGCs that are not expressed under standard laboratory conditions [147,168]. Whole-genome sequencing and bioinformatic tools like antiSMASH [169] allow for the systematic identification of these clusters. Subsequent activation employs innovative strategies, including: (i) heterologous expression in optimized bacterial hosts [170]; (ii) promoter and ribosome engineering to dysregulate cellular metabolism; and (iii) co-cultivation to simulate ecological competition [171].

II. Exploring Unexplored Ecological Niches and the Microbiome

Building on the success of bioprospecting in mangroves and marine sediments, future discovery pipelines must prioritize ecological intelligence. This entails systematically targeting underexplored extreme environments (hypersaline lakes, deep-sea vents, and deserts) [172] and delving into the complex chemical interactions within host-associated microbiomes [173].

III. Leveraging Artificial Intelligence and Machine Learning

The vast amount of genomic and metabolomic data is ripe for analysis by AI. Machine learning models can predict BGCs with higher accuracy, link genomic sequences to chemical structures, and even predict the bioactivity and toxicity of novel metabolites prior to isolation, dramatically accelerating lead prioritization [174,175,176].

IV. Combinatorial Biosynthesis and Synthetic Biology

Beyond discovering new scaffolds, we can now engineer them. Combinatorial biosynthesis involves mixing and matching enzymatic domains from different BGCs to create “non-natural” natural products with optimized properties, such as enhanced potency or the ability to overcome specific resistance mechanisms [177,178]. Synthetic biology allows for the refactoring of BGCs for efficient expression and the de novo creation of novel antibiotic pathways.

V. Integrating Metabolomics and Advanced Analytics

Rapid dereplication is critical for efficiency. High-resolution mass spectrometry and NMR-based metabolomics, coupled with platforms like Global Natural Products Social Molecular Networking (GNPS), enable the rapid comparison of metabolite profiles across samples, ensuring focus on truly novel chemistries [179,180].

Ultimately, unlocking the full anti-MRSA potential of *Actinomycetota* demands an integrated strategy that merges ecological intelligence with cutting-edge technology. A synergistic framework, combining targeted bioprospecting in underexplored niches, deep-genome mining, and synthetic biology, is essential to systematically access this vast, untapped chemical landscape. By bridging natural product discovery with translational science, we can accelerate the delivery of novel antimicrobial scaffolds. The path forward therefore requires a concerted effort to mine the chemical arsenal of *Actinomycetota* through the integrated strategies outlined herein. The validity and broad applicability of this ecology- and genomics-driven paradigm are powerfully demonstrated by its pivotal role in yielding therapeutic leads for other therapeutically challenging diseases, such as tuberculosis [181] and cancer [182,183]. This track record provides a compelling blueprint and strong justification for its continued and expanded application in the urgent quest for novel anti-MRSA agents and other future medicines.

## Figures and Tables

**Table 1:** Secondary metabolites produced by *Actinomycetota* with reported activity against MRSA.

Compound	*Actinomycetota* Species	Isolation Source	MIC (μg/mL)	MIC (μM)	MRSA Strain	Country	Reference
Actinomycin D	*Streptomyces* sp. ZZ338	Rocks	0.08	0.06	ATCC 43300	CN	[62]
Actinomycin V			0.08	0.06			
Actinomycin X_0_β			0.61	0.48			
Actinomycin X_2_	*Streptomyces globisporus* WA5-2-37	Animal-associated (*Periplaneta americana*)	0.25	0.20	ATCC 43300	CN	[63]
Actinomycin X_2_	*Streptomyces smyrnaeus* UKAQ_23	Mangrove	3.13–12.5	2.46	Clinical isolate	AS	[64]
Actinomycin D			12.5–25	9.96			
Actinomycin V	*Streptomyces* sp. M7	Soil	3.95	3.11	Clinical isolate	IN	[65]
Actinomycin X_2_			3.50	2.76			
Actinomycin D			4.0	3.19			
Fijimycin A	*Streptomyces* sp. CNS-575	Marine sediments	4–32	4.55	Clinical isolate	FJ	[66]
Fijimycin B			>32	37.25			
Fijimycin C			8–32	8.94			
Etamycin A			4–16	4.55			
Diperamycin	*Streptomyces griseoaurantiacus* MK393-AF2	Soil	0.10	0.12	Clinical isolate	JP	[67]
Vinylamycin	*Streptomyces* sp. MI982-63F1	Soil	3.13	6.34	Clinical isolate	JP	[68]
Lunaemycin A	*Streptomyces lunaelactis* MM109 T	Moonmilk deposits	0.12	0.17	ATCC 43300	BE	[69]
NW-G01	*Streptomyces alboflavus* 313	Soil	7.82	10.33	Clinical isolate	CN	[70]
NW-G08	*Streptomyces alboflavus* 313	Soil	1.56	2.02	Clinical isolate	CN	[71]
NW-G09			12.5	16.16			
Zunyimycin A	*Streptomyces* sp. FJS31-2	Soil	6.9–16.7	13.02	Clinical isolates	CN	[72]
Zunyimycin B			7.9–25.6	14.92			
Zunyimycin C			3.8–8.1	6.68			
Medermycin	*Streptomyces albolongus* CA-186053	Animal-associated (marine sponge)	2.0	4.37	Clinical isolate	GQ	[73]
G-15F			4.0	8.42			
Strepoxepinmycin A	*Streptomyces* sp. XMA39	Marine sediments	12	25.3	ATCC 43300	CN	[74]
Strepoxepinmycin B			15	29.7			
Strepoxepinmycin C			6	11.8			
Strepoxepinmycin D			3	6.36			
Medermycin			0.25	0.55			
Streptovertimycin A	*Streptomyces morookaense* SC1169	Soil	2.50	4.80	Clinical isolate	CN	[75]
Streptovertimycin B			2.50	4.50			
Streptovertimycin C			2.50	4.50			
Streptovertimycin D			1.25	2.31			
Streptovertimycin E			2.50	4.34			
Streptovertimycin F			2.50	4.34			
Streptovertimycin G			0.63	1.07			
Streptovertimycin H			5	8.19			
Streptovertimycin U	*Streptomyces morookaense* SC1169	Soil		2.50	Clinical isolate	CN	[76]
Streptovertimycin V				>10			
14-Bromo-streptovertidione				>10			
Streptovertimycin W				>10			
Streptovertimycin X				5			
Streptovertimycin Y				>10			
Streptovertimycin Z1				>10			
Streptovertimycin Z2				5			
Streptovertimycin Z3				1.3			
Streptovertimycin Z4				1.3			
Streptovertimycin Z5				1.3			
Fasamycin R				2.50			
Fasamycin S				0.6			
Accramycin A				1.3			
Accramycin B				>10			
Streptorubin B	*Streptomyces* sp. MC11024	Soil	32	81.7	Clinical isolate	JP	[77]
8-O-methyltetrangomycin	*Streptomyces* sp. SBRK2	Animal-associated (*Spirostella* sp.)	2	5.95	Clinical isolate	IN	[78]
Lactoquinomycin A	*Streptomyces bacillaris* MBTC38	Marine sediments	0.06–0.25	0.13	ATCC 43300 and clinical isolates	KR	[79]
Lactoquinomycin B			1–8	2.11			
N-methyl actoquinomycin A			0.25–1	0.56			
Menoxymycin A			0.5–2	1.06			
Neoabyssomycin F	*Streptomyces koyangensis* SCSIO 5802	Marine sediments	16	22.0	Clinical isolate	CN	[80]
Neoabyssomycin G			16	22.0			
Weddellamycin	*Streptomyces* sp. DSS69	Animal-associated (marine sponge)	0.10	0.23	Clinical isolate	AQ	[81]
Polyketomycin	*Streptomyces* sp. MK277-AF1	Soil	0.2	0.23	Clinical isolate	JP	[82]
Citreamicin θA	*Streptomyces caelestis*	Sea water	0.25	0.43	ATCC 43300	AS	[83]
Citreamicin θB			0.25	0.43			
Citreaglycon A			8	15.4			
Dehydrocitreaglycon A			0.25	0.50			
Lactonamycin	*Streptomyces rishiriensis* MJ773-88K4	Soil	0.39–0.78	0.68	Clinical isolate	JP	[84]
N-acetyl-N-demethylmayamycin	*Streptomyces* sp. 182SMLY	Marine sediments	10	20.35	ATCC 43300	CN	[85]
Waldiomycin	*Streptomyces* sp. MK844-mF10	Soil	16	22.8	Clinical isolate	JP	[86]
Isoikarugamycin	*Streptomyces zhaozhouensis* CA-185989	Marine sediments	2–4	4.18	Clinical isolate	GQ	[87]
28-N-methylikarugamycin			1–2	2.03			
Ikarugamycin			2–4	4.18			
Albocycline	*Streptomyces* sp. 6–31	Soil	0.5–1	1.62	Clinical isolate	JP	[88]
11′,12′-dehydroelaiophylin	*Streptomyces* sp. 7–145	Marine sediments	2	1.99	ATCC 33591	CN	[89]
11,11′-O-dimethyl-14′-deethyl-14′-methylelaiophylin			32	30.8			
Elaiophylin			1	0.98			
11-O-methylelaiophylin			2	1.92			
11,11′-O-dimethylelaiophylin			16	15.19			
Efomycin G			2	1.98			
Quadoctomycin	*Streptomyces* sp. MM168-141F8	Soil	1–2	0.70	Clinical isolate	JP	[90]
30-demethyllydicamycin	*Streptomyces platensis* TP-A0598	Sea water	3.13	3.72	Clinical isolate	JP	[91]
14,15-dehydro-8-deoxylydicamycin			6.25	7.47			
30-demethyl-8-deoxylydicamycin			1.56	1.89			
8-deoxylydicamycin			3.13	3.73			
Bafilomycin D	*Streptomyces* sp. HZP-2216E	Plant-associated (*Ulva pertusa*)	33.1	54.7	ATCC 43300	HT	[92]
9-hydroxybafilomycin D			33.2	53.5			
Bafilomycin A1			16.8	27.0			
23-O-butyrylbafilomycin D			7.4	11.0			
Hygrocin N	*Streptomyces* sp. ZZ1956	Mangrove	15	30.5	Clinical isolate	IR	[93]
Hygrocin O			24	47.2			
Hygrocin R			9	17.2			
Hygrocin T			44	91.6			
Hygrocin U			3	10.6			
2-amino-6-hydroxy-7-methyl-1,4-naphthoquinone			10	49.3			
2-acetamide-6-hydroxy-7-methyl-1,4-naphthoquinone			3	12.3			
3′-methoxy(1,1′,4′,1″-terphenyl)-2′,6′-diol			5	17.1			
Echoside C			6	13.2			
Echoside A			8	17.1			
AN483	*Streptomyces* sp. AN100483	Soil	32	100.41	Clinical isolate	KR	[94]
2,3-dihydroxy-9,10-anthraquinone	*Streptomyces galbus* ERINLG-127	Soil	12.5	52.07	Clinical isolate	IN	[95]
Napyradiomycin A3	*Streptomyces* sp. CA-271078	Soil	>96	>217.0	Clinical isolate	TN	[96]
Napyradiomycin B7a			48	96.5			
Napyradiomycin B7b			>64	>128.7			
Napyradiomycin SC			>96	>186.3			
Napyradiomycin D1			12–24	25.0			
MDN-0170			>96	>200.4			
3-chloro-6, 8-dihydroxy-8-α-lapachone			48–96	155.5			
3-chloro-6-hydroxy-8-methoxy-α-lapachone			>64	>198.7			
Napyradiomycin B6			48–96	96.4			
18-hydroxynapyradiomycin A1			48–96	96.5			
Napyradiomycin A2a			12–24	24.2			
Napyradiomycin A2b			12–24	24.2			
Napyradiomycin B4			12–24	22.5			
Napyradiomycin B2			3–6	6.29			
Napyradiomycin B5			12–24	23.0			
Napiradiomycin 1	*Streptomyces* sp. CNH-070	Marine sediments	16	34.9	Clinical isolate	US	[97]
Napiradiomycin 2			64	145.4			
Napiradiomycin 3			>64	>139.7			
Napiradiomycin 4			>64	>134.4			
Napiradiomycin 5			>64	>122.6			
Napiradiomycin 6			>64	>128.5			
Napiradiomycin B2			32	67.1			
Napiradiomycin B3			2	3.58			
Napiradiomycin B4			32	59.9			
MDN-0170	*Streptomyces* CA-271078	Marine sediments	>64	133.6	Clinical isolate	ST	[98]
4-dehydro-4a-dechloronapyradiomycin A1			4–8	8.99			
Napiradiomycin A1			0.5–1	1.03			
3-chloro-6,8-dihydroxy-8-α-lapachone			>64	>200			
Streptoglutarimide A	*Streptomyces* sp. ZZ741	Marine sediments	9	30.5	Clinical isolate	CN	[99]
Streptoglutarimide B			11	37.3			
Streptoglutarimide C			10	34.1			
Streptoglutarimide D			10	33.9			
Streptoglutarimide E			9	28.9			
Streptoglutarimide F			10	32.1			
Streptoglutarimide G			10	33.9			
Streptoglutarimide H			9	30.3			
Streptoglutarimide I			11	37.0			
Streptoglutarimide J			10	35.8			
Streptovitacin A			10	33.6			
Collismycin A	*Streptomyces globisporus* WA5-2-37	Animal-associated (*Periplaneta americana*)	8	29.1	ATCC 43300	CN	[63]
Antibiotic E-975	*Streptomyces* sp. AT37	Soil	20	49.0	Clinical isolate	DZ	[100]
Cremimycin	*Streptomyces* sp. MJ635-86F5	Soil	0.39–0.78	0.62	Clinical isolate	JP	[101]
Chromomycin A9	*Streptomyces microflavus* MBTI36	Marine sediments	0.13	0.11	ATCC 43300, ATCC 700787, ATCC 700788 and clinical isolate	KR	[102]
Chromomycin Ap			0.06–0.25	0.05			
Chromomycin A2			0.06–0.25	0.05			
Chromomycin A3			0.13	0.11			
1-Monolinolein	*Streptomyces* sp. YBQ59	Plant-associated (*Cinnamomum cassia*)	8.5	24.0	ATCC 35984	VN	[103]
Bafilomycin D			11.1	18.4			
Nonactic acid			18.6	92.0			
Daidzein			24.8	97.6			
3′-Hydroxydaidzein			36.1	133.6			
Nosokomycin A	*Streptomyces* sp. K04-0144	Soil	0.125	84.0	Clinical isolate	JP	[104]
Nosokomycin B			0.125	84.1			
Nosokomycin C			0.125	94.3			
Nosokomycin D			0.125	94.2			
Plicacetin	*Streptomyces* sp. SP5	Soil	3.8	7.3	Clinical isolate	JP	[105]

Two-letter country codes defined in ISO 3166-1 [106]. NA: not available.

**Table 2:** Crude extracts/unidentified compounds isolated from *Actinomycetota*.

Crude Extracts/Unidentified Compounds	*Actinomycetota* Species	Isolation Source	MIC/Disk Diffusion	MRSA Strain	Country	Reference
Ethyl acetate fraction	*Streptomyces* sp. VITBRK2	Marine sediments	17 mm	ATCC 29213	IN	[107]
Dichloromethane fraction	*Streptomyces* sp. M10-77	Marine sediments	40 mm	ATCC 43300	PE	[108]
Methanolic fraction	*Streptomyces* SMC 277 ^T^	Soil	9.3 mm	Clinical isolate	TH	[109]
Antibiosis test (agar-plug test)	*Streptomyces* sp. EMB24	Soil	22 mm	ATCC 43300	IN	[110]
Antibiosis test (agar-plug test)	*Streptomyces* sp. MUSC 135 T e MUSC 137T	Soil	10.5 mm	ATCC BAA-44	MY	[111]
Ethyl acetate fraction	*Streptomyces californicus*	Plant-associated (*Datura metel*)	21.3 mm	ATCC 43300	IN	[112]
Ethyl acetate fraction	*Streptomyces* NIOT-Ch-40	Marine sediments	1.56 μg/mL	Clinical isolate	BD	[113]
Ethyl acetate fraction	*Streptomyces griseoplanus* NRRL-ISP 5009	Soil	2.5 µg/mL	Clinical isolate	NG	[114]
Ethyl acetate fraction	*Streptomyces cavourensis* MH16	Plant-associated (*Millingtonia hortensis*)	25 μg/mL	ATCC 33915	IN	[115]
Ethyl acetate fraction	*Streptomyces* sp. SUK 25	Plant-associated (*Zingiber spectabile*)	1.95 µg/mL	ATCC 49476	MY	[116]
Ethyl acetate fraction/Unidentified compound	*Streptomyces pharmamarensis* ICN40	Animal-associated (marine sponge)	>10 mm	ATCC 33591	IN	[117]
Ethyl acetate fraction/Unidentified compounds	*Streptomyces* sp. CS392	Soil	2.03–4.06 µg/mL	Clinical isolate	KR	[118]
Methanolic fraction	*Streptomyces* sp. O PVRK-1	Soil	32–34 µg/mL	Clinical isolate	IN	[119]
Ethyl acetate fraction	*Streptomyces* sp. MNP32	Soil	12 μg/mL	Clinical isolate	IN	[120]
Ethyl acetate fraction	*Streptomyces* sp. 4054	Marine sediments	12 mm	ATCC 33591	BR	[121]
Ethyl acetate fraction/Unidentified compound	*Streptomyces* sp. O MN41	Marine sediments	2.8 µg/mL	ATCC 33591	IR	[122]
Chloroformic fraction	*Streptomyces* sp. JRG-02	Soil	1.25 μg/mL	Clinical isolate	IN	[123]
Methanolic fraction/Unidentified compound	*Streptomyces rubrolavendulae* ICN3	Soil	2.5 μg/mL	Clinical isolate	IN	[124]
Ethyl acetate fraction	*Streptomyces* sp. BT-408	Marine sediments	64 µg/mL	ATCC 33591	IN	[125]

## Data Availability

The original contributions presented in this study are included in the article. Further inquiries can be directed to the corresponding author.
